# Clinical Characteristics and Treatment of Intra-abdominal Aggressive Fibromatosis: A Retrospective Study of 16 Patients

**DOI:** 10.3389/fmed.2020.00002

**Published:** 2020-01-31

**Authors:** Jianchun Xiao, Jinzhu Mao, Binglu Li

**Affiliations:** Department of General Surgery, Peking Union Medical College Hospital, Chinese Academy of Medical Sciences, Beijing, China

**Keywords:** aggressive fibromatosis, FAP, recurrence, surgery, systemic therapy

## Abstract

**Background:** This study aimed to investigate the clinical characteristics and treatment methods for intra-abdominal aggressive fibromatosis.

**Methods:** We reviewed the clinical data from 16 patients who were diagnosed with intra-abdominal aggressive fibromatosis and were admitted to Peking Union Medical College Hospital between March 1983 and September 2018.

**Results:** Among the 16 patients, 11 patients presented with a hard smooth abdominal mass with clear borders and a diameter of 4.3–25.0 cm. Six patients had a history of abdominal surgery and 3 patients had a history of familial adenomatous polyposis. Computed tomography imaging revealed a slightly dense mass with mild-to-moderate enhancement. Of all the 16 patients, 11 patients underwent surgical treatment and no recurrence occurred in 10 case after complete resection while recurrence occurred in 1 case after partial resection. Two patients underwent surveillance and 3 patients received cytotoxic drugs treatment, and no disease progression was observed via imaging during their follow-up.

**Conclusions:** Intra-abdominal aggressive fibromatosis is histologically benign tumor with high local recurrence rate. Surgery is an effective treatment and complete resection is essential in reducing the local recurrence rate.

## Introduction

Aggressive fibromatosis (AF), which is also known as desmoid tumors (DTs) or desmoid-type fibromatosis (DF), is a rare benign monoclonal fibroblastic proliferation that is derived from muscle, tendon or ligament tissue. This growth is characterized by local infiltration, high recurrence rate and no metastasis (1, 2). Furthermore, AF can occur in any part of the body, including the extremities, trunk and abdomen (1), and AF is clinically categorized according to the site of development as extra-abdominal AF, abdominal AF or intra-abdominal AF (3). Among these categories, intra-abdominal AF has the lowest incidence and the poorest prognosis, and its diagnosis and treatment remain challenging.

AF can be divided into sporadic and hereditary cases, with sporadic cases accounting for the highest proportion of new AF cases that occur at an annual rate of ~4–6 cases/1,000,000 population (1). Sporadic cases are related to trauma, surgery or estrogen status/treatment, while hereditary cases are related to familial adenomatous polyposis (FAP) or adenomatous polyposis coli (APC) mutations (4). It is estimated that ~5–10% AF cases arise in patients with FAP, while ~10–20% AF cases develop FAP (1, 5). Mutation of β-catenin mutation is observed in 85–90% AF cases, which is significantly correlated with an increased risk of post-operative recurrence (2). Furthermore, APC mutations are biomarker of FAP and are observed in 16% pediatric patients with AF (6). However, it is interesting that β-catenin and APC mutation are exclusive in AF (2).

Considering that the biological behaviors and clinical features of intra-abdominal AF are poorly understood, we retrospectively reviewed clinical data from 16 patients with intra-abdominal AF to summarize their clinical characteristics, treatment strategies and outcomes.

## Materials and Methods

This retrospective study evaluated 16 Chinese patients who were diagnosed with intra-abdominal AF and admitted to Peking Union Medical College Hospital (PUMCH) between March 1983 and September 2018. The patients' medical records included demographic characteristics (sex and age), clinical findings (manifestations, laboratory examination, and radiological findings), treatment regimens (surveillance, surgery, and systemic therapy), and follow-up results (manifestations and recurrence). Laboratory tests included tumor antigen, such as CEA and CA19-9. Imaging findings included the location, size and number of tumors, as well as blood flow information and enhancement status when available. The pathological records included the tumor size, gross macroscopic findings and immunohistochemical findings. The clinical diagnosis of intra-abdominal AF was based on the manifestations and radiological findings, while the definite diagnosis of intra-abdominal AF was based on the histopathological results. This retrospective study did not directly involve patient subjects and was approved by the Ethics Review Committee of PUMCH.

The statistical analyses were performed using IBM SPSS (version 22.0; IBM Corp., Armonk, NY, USA). Categorical variables were summarized as number (percentage) and were analyzed using the Chi-square test or Fisher's test. Log-rank test was used to evaluate survival differences. *P* < 0.05 was considered statistically significant.

## Results

### Clinical Data

The clinical and demographic characteristics of 16 patients with intra-abdominal AF admitted to PUMCH between September 1983 and September 2018 are shown in Table 1. The 16 patients included 8 men and 8 women, and the mean age was 39 years. Eleven patients presented with an abdominal mass, 3 patients presented with abdominal pain, and 2 patients presented with no discomfort. The physical examination revealed an abdominal mass in 12 patients, and 2 patients had a large mass with mild tenderness. The abdominal masses ranged from 4.3 to 25 cm (Table 1), and were hard and smooth with clear borders. All patients had no symptoms of fever, fatigue and weight loss. Three patients had previously undergone total colorectal resection for FAP and had received six 3-week cycles of oxaliplatin plus capecitabine, based on focal carcinogenesis that was detected during the pathologic examination. These 3 cases experienced recurrence, with an 8 cm mass detected after 12 months, a 5 cm mass detected after 14 months, and a 10 cm mass detected after 24 months. Four patients had had previously given birth, including 1 case involving a cesarean section. One patient had undergone appendectomy and one patient had undergone splenectomy plus pancreatectomy. One patient had undergone partial resection twice (3 years apart) as previous treatment for the intra-abdominal AF, and the remaining 15 patients had not been previously treated for their intra-abdominal AF.

**Table 1:** Clinical data from the 16 cases of intra-abdominal aggressive fibromatosis.

**Clinical characteristics**	**Number (%)**
**Sex**	
Male	8 (50%)
Female	8 (50%)
Mean age (years)	39
**Manifestations**	
Abdominal mass	11 (68.75%)
Abdominal pain	3 (18.75%)
No discomfort	2 (12.5%)
**Tumor size**	
<5 cm	1 (6.25%)
5–10 cm	9 (56.25%)
10–15 cm	4 (25%)
15–20 cm	1 (6.25%)
≥20 cm	1 (6.25%)
**Medical history**	
Total colorectal resection	3 (18.75%)
Cesarean section	1 (6.25%)
Splenectomy and pancreatectomy	1 (6.25%)
Appendectomy	1 (6.25%)
**Precious treatment**	
Surgery	1 (6.25%)
None	15 (93.75%)

**Figure 1:**
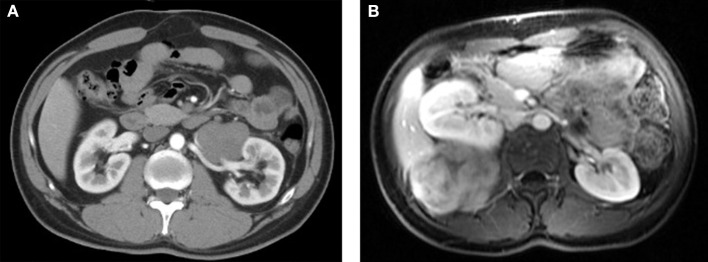
**(A)** Computed tomography revealed a low-density mass around the left renal artery. **(B)** Magnetic resonance imaging revealed an isointense mass with a hyperintense signal at the right lumbar psoas muscle.

Abdominal ultrasonography was performed for 7 patients, which revealed an oval, smooth, solid mass with clear borders, and hypoechogenicity. Three of these cases exhibited rich in blood flow signals. Twelve patients underwent computed tomography (CT), which revealed an oval or irregular, slightly rough, slightly dense soft-tissue mass with mild-to-moderate enhancement (Figure 1A). One patient underwent magnetic resonance imaging (MRI), which revealed an isointense signal on T1-weighted images (T1WI) and a heterogeneous hyperintense signal on T2-weighted images (T2WI), with significant enhancement in the arterial phase and delayed enhancement in the portal venous phase (Figure 1B). Digital subtraction angiography (DSA) was performed for 3 patients, which revealed abundant blood flow from the internal iliac artery to the tumor in 2 cases. Four patients underwent tumor biopsy, and microscopic examination revealed fusiform cells. Serological tests for CEA and CA19-9 were performed for 7 patients, although no abnormal findings were observed.

### Management Strategies

The management strategies involved surveillance for 2 patients, surgery for 11 patients and cytotoxic drug treatment for 3 patients (Table 2). The 2 patients chose surveillance because the mesenteric vessels were surrounded by the masses, which would have made complete resection difficult. One of these patients was diagnosed with intra-abdominal AF based on biopsy, and selected surveillance plus pain management using non-steroidal anti-inflammatory drugs (NSAIDs) and morphine. In the other case, imaging revealed multiple intra-abdominal masses close to the left renal blood vessels and the inferior vena cava, which would have complicated surgery and potentially required left kidney resection. The patient chose surveillance because there were no discomfort or renal dysfunction.

**Table 2:** Treatments for the 16 cases of intra-abdominal aggressive fibromatosis.

**Strategy**	**Number (%)**
Surveillance	2 (12.5%)
Surgery	11 (68.75%)
**Tumor location**	
Mesentery	5
Retroperitoneum	6
**Involvement**	
Superior mesenteric artery	2
Abdominal aorta	1
Iliac vessels	1
Inferior vena cava	1
Renal vessels	1
Ureter or bladder	4
Intestine and colon	4
Psoas muscle	1
Bone	2
**Resection margin**	
R0	10
R1	0
R2	1
Radiotherapy	0 (0)
**Systemic therapy**	
Cytotoxic drugs	3 (18.75%)

Among the 11 surgically treated cases, 1 patient underwent laparoscopic surgery, and 10 cases were treated via laparotomy. Five masses were located at the mesentery and 6 masses were located at the retroperitoneum, with diameters ranging from 5 to 20 cm in diameter. Organs involvement was observed in 9 cases, including 6 cases with single-organ involvement, and 3 cases with multiple-organs involvement. The affected tissues or organs included the mesentery, superior mesenteric artery, abdominal aorta, inferior vena cava, external iliac vein, jejunum, colon, ureter, bladder, psoas muscle, lumbar vertebrae, and ilium. Complete resection was performed in 10 cases and partial resection was performed in 1 case. In the partial resection case, a 10 cm mass surrounded the mesenteric vessels and partial resection had previously been attempted twice 3 years before administration. At the second recurrence, we observed a 12 × 11 × 10 cm mass surrounding the superior mesenteric artery, abdominal aorta and vena cava. Since complete resection would have been difficult and might have impaired intestinal function, we attempted partial resection to alleviate tumor progression.

The 3 patients who received cytotoxic drugs treatment (gemcitabine in 3-week cycle) had been diagnosed with intra-abdominal AF at 2 years after total colectomy for FAP, which was followed by treatment using oxaliplatin plus capecitabine. Considering their surgery history and lack of discomfort, these patients selected drug treatment instead of surgery. All patients underwent regular radiographic assessments to monitor for post-treatment tumor growth.

### Follow-Up

The follow-up time ranged from 4 to 72 months (average: 23 months), which involved imaging assessments every 6 months using ultrasonography, CT and MRI (Table 3). For the 2 cases that involved surveillance, the abdominal pain was well-controlled by the combination of NSAIDs and morphine in 1 case, with the tumor size growing slightly from 4 to 4.2 cm from in diameter after 6 months. In the other case, the patient reported no discomfort and the mass size remained stable for 36 months. Both patients continued to undergo active surveillance.

**Table 3:** Follow-up statuses for patients who received different treatments.

**Treatment**	**No**.	**Strategy**	**Follow-up status**
Surveillance	2	1/2: NSAIDs + morphine	The mass grew from 4 to 4.2 cm over a 6-month period.
		1/2: surveillance	The mass remained stable over a 36-month period.
Surgery	11	3/11: partial resection	Recurrence after 6 years.
		8/11: complete resection	No recurrence.
Systemic therapy	3	3/3: gemcitabine	At 6 months, the three cases involved a stable mass, shrinkage from 5 to 3 cm, and shrinkage from 10 to 8 cm.

For the 11 surgically treated cases, pathological examination confirmed the diagnoses of intra-abdominal AF. During every 6 months follow-up, the 10 cases that involved complete resection recovered well and did not exhibit recurrence. The patient who underwent partial resection experienced a third recurrence after 6 year. The CT findings revealed multiple giant soft-tissue masses (12 × 13 cm) surrounding the superior mesenteric artery and compressing the surrounding organs. To alleviate abdominal pain and the tumor burden, a third partial resection was attempted, although we lost the patient to follow-up after 6 months.

The 3 patients who received gemcitabine treatment underwent follow-up at 6-month intervals. After the first follow-up, the mass remained stable at 8 cm in the first case, the mass shrank from 5 to 3 cm in the second case and the mass shrank from 10 to 8 cm in the third case. All 3 patients decided to continue the gemcitabine treatment.

## Discussion

AF are rare and involve a monoclonal fibroblastic proliferation derived from deep connective or muscle tissue, which is characterized by a benign pathology, invasive growth, a high local recurrence rate and no metastasis (1, 7). While AF can affect almost all parts of the body, the most commonly affected locations are the abdominal wall, neurovascular bundle of the extremities, the mesenteric root, and the head and neck. The incidence of AF is very low (~4–6 cases/1,000,000 population per year) and accounts for only 0.03% of all tumors and 3% of soft tissue tumors (2, 4). AF commonly develops between the age of 15 and 60 years (average: 30 years) (8) and it is more likely to involve female patients (male:female ~1:3) (9). The 5-year recurrence is ~50%, and recurrence is related to age, tumor location and resection margin status (10). Intra-abdominal AF is the least common subtype (~15%) and typically involves the small mesentery and retroperitoneum, with multiple lesions observed in ~10% cases (1). In our patients, the male:female ratio was 1:1 and the median age was 39 years, which was consistent with the lack of a sex-based prevalence in older patients (1). The tumors developed in the small mesentery (5 cases), the retroperitoneum (10 cases), or both sites (1 case), and 4 cases exhibited multiple lesions.

The etiology and pathogenesis of AF remain unclear and are possibly related to 3 factors. The first set of factors is genetic, as ~5–15% AF cases will develop FAP, especially in patients who are male, <60 years old and have a lesion located at the abdominal wall or in the abdomen. Furthermore, ~10–20% FAP cases will develop AF (Gardner syndrome), and patients with FAP are >800× more likely to develop AF, relative to the general population. Moreover, the Gardner syndrome is associated with a higher local recurrence rate than the other AFs (4, 5). Overexpression and accumulation of β-catenin can activate fibroblastic proliferation signaling and induce tumorigenesis as a result of mutation in the APC or β-catenin gene on chromosome 5q21-q22 (11). In the present study, 3 of the 16 patients (18.75%) had a history of FAP and their intra-abdominal AF occurred within 2 years after total colorectal resection for the FAP, which suggested that FAP was potentially involved in their development of AF. However, we are unable to comment on their status regarding β-catenin or APC mutations, as the relevant data were not collected. The second set of factors is trauma or surgery, as ~30% patients with AF have a history of trauma (12), which indicates that the wound healing process may induce fibroblastic proliferation and promote AF formation. In the present study, 6 of the 16 patients (37.5%) had a history of abdominal surgery, which is consistent with the previous report. The third set of factors is estrogen status and/or related treatment. For example, the relationship between high estrogen levels and AF has been observed in many cases, although the underlying mechanism remains unclear (9). Pregnancy can also increase the incidence of AF, especially for women who undergo a cesarean section, although the prognosis of pregnancy-related AF is better than that of the other types. Furthermore, treatment using estrogen, progesterone and androgen receptor blockade can induce tumor regression.

The manifestations of AF are variable, not specific, and are related to the affected site (7). In many cases, the AF undergoes as asymptomatic development and chronic progression, leading to a palpable solid abdominal mass that may be associated with abdominal pain. Gastrointestinal obstruction, perforation and symptoms of specific organs involvement appear in some severe cases. In some cases, the AF may stabilize or regress over time, with ~20% AF cases exhibiting natural regression (13). In our cohort, 1 patient experienced a 36-month period without discomfort, renal dysfunction, or tumor growth (6.3 × 4.8 cm, close to the left renal hilum). Relative to the other cases, especially the case with recurrence, that patient's stable clinical course may reflect the tumor's intrinsic characteristics, which may indicate that the clinical behaviors of intra-abdominal AFs are heterogenous. This variable clinical course may also indicate that non-surgical treatment strategies can be considered in select cases.

Ultrasonography, CT and MRI are the most common modalities for assessing AF cases. Ultrasonography is inexpensive, rapid and widely used as a diagnostic modality, which typically reveals the AF as a round or oval, smooth and solid soft-tissue mass with variable echogenic components. The cellular components usually exhibit hyperechogenicity, while the matrix and fibrous components usually exhibit hypoechogenicity. The utility of a Doppler sequence will depend on the tumor's vascularity (14). However, considering its lack of specificity, ultrasonography is typically only used as a primary examination and is not recommended as a monitoring modality. Relative to ultrasonography, CT and MRI can provide more detailed anatomical information, which is critical for assessing tumor resectability and planning any surgical treatment. The first choice for diagnosis and evaluation is typically CT, especially for intra-abdominal AF, which generally reveals a soft-tissue mass that is slightly denser than the skeletal muscle, with intra-tumor collagen components that can exhibit high density. Mild-to-moderate enhancement can be observed, while necrosis and calcification are rare (14). Twelve of our patients underwent CT, which revealed the typical appearance of a slightly dense soft-tissue mass with mild enhancement. Relative to CT, MRI provides better resolution of the soft tissue and is recommended for evaluations in cases involving extra-abdominal AF and recurrence (15). Heterogeneous MRI signals are commonly observed because the AF contains spindle cells, collagen fibers and extracellular matrix, which typically exhibit an isointense signal on T1WI and a hyperintense signal on T2WI. Richer cellular components are associated with higher signal. There is also a significant enhancement in the arterial phase and delayed enhancement in the portal venous phase, which is more obvious when the tumor has a higher proportion of cellular components (16, 17). In addition, the response to systemic therapy can be predicted by monitoring the tumor size and any decrease in the SUV value based on FDG-PET/CT, while MRI can also be used to monitor the tumor size and change in the T2WI signal or the degree of enhancement decrease (1, 14, 18).

During the last decade, surgery alone and surgery plus radiotherapy were the most common strategies for managing AF. However, the unique biological behavior of AF has indicated that these tumors may remain stable or undergo a natural regression. Furthermore, the long-term local recurrence rate is high after surgical resection. Therefore, a new management model has been suggested, which involves systemic treatment based on active surveillance, with surgery and radiotherapy serving as local treatment for symptom control (4, 14, 19). The main management strategies are described follow.

### Surveillance

Approximately 50% patients with AF may have a stable disease or experience natural regression, which has led many clinicians to recommend active surveillance as the primary management strategy (7). For patients with no symptoms, mild symptoms or mild progression based on imaging, a surveillance duration of 12–24 months is may be appropriate, with symptomatic treatment as necessary such as pain management (2). In this context, one cohort study of 771 patients found no significant difference in overall survival between the surgery and non-surgery groups (20). Two of our patients chose surveillance, which did not identify any disease progression during their follow-up. Nevertheless, larger and longer studies are needed to examine the long-term outcomes observed during surveillance.

### Surgery

Surgical treatment remains the first-line strategy, especially for patients with severe symptoms, rapid progression, important organ involvement, or serious complications (7). Complete resection is recommended and ensuring negative margins can reduce the local recurrence rate (4). The 3-year local recurrence rate after surgery is ~40–50% (21), with long-term local recurrence rate of ~25–70% (22), although negative margins are associated with a much lower recurrence rate relative to positive margins (~10 vs. 80%) (23). Nevertheless, local recurrence seems to be a biological event that does not affect the overall survival rate. Therefore, surgical techniques that preserve organ function and ensure the maximal survival benefit may be superior to complete resection (4). Among our 11 patients who underwent surgery, 10 patients did not experience recurrence after complete resection, although the patient who underwent partial resection experienced recurrence after 6 years, which further suggests that the surgical margin status is related to the recurrence rate.

### Radiotherapy

Radiotherapy is primarily recommended for symptomatic patients with contraindications for surgery or systemic therapy, such as very old patients (7, 19). Nuyttens et al. performed a retrospective study that revealed that radiotherapy or radiotherapy after surgery achieved a local control rate of up to 70%, which was significantly higher than the rate for surgery alone, regardless of margin and recurrence (24).

### Systemic Therapy

Systemic therapy can involve hormonal therapy, cytotoxic drugs therapy, targeted therapy or some novel drugs. Although the mechanism of systemic therapy in this setting remains poorly understood, its efficacy can reach 40% and it can reduce the local recurrence rate (25). Hormone therapy can involve androgen antagonists or progestins and can be used for patients with less aggressive AF (7). Androgen antagonists, such as tamoxifen and toremifene, can also inhibit tumor growth by regulating TGF-β and β-catenin signaling, which inhibits the activation of fibroblastic proliferation (11). Previous research has recommended toremifene as the first choice, with non-FAP-related cases reportedly responding better than FAP-related cases. Chemotherapy may be more appropriate for aggressive intra-abdominal AF, especially the FAP-related type (26), which may be because it provides more effective and durable cytoreduction (27, 28). Therefore, hormone therapy can be used as the primary choice for systemic treatment, with other strategies considered if hormone therapy provides poor efficacy. Cytotoxic drug therapy (e.g., liposomal doxorubicin, methotrexate, and vinorelbine) is often used in patients with obvious symptoms, rapid progression, or important organ involvement (7), with the recommended regimen involving low-dose methotrexate and/or vinblastine or anthracyclines (1). Our 3 patients with FAP-related intra-abdominal AF received second-line gemcitabine treatment based on pathological evidence of local carcinogenesis and the desmoid tumor developed after the first-line treatment using oxaliplatin plus capecitabine. These 3 cases had previously undergone total colorectal resection and the decision to not perform a second surgery was based on their physical condition and willingness, despite surgery being the main strategy for AF. These 3 cases revealed that systemic therapy using cytotoxic drug was effective for controlling the growth of FAP-related intra-abdominal AFs, providing additional information regarding the potential utility of systemic therapy in this setting. Tyrosine kinase inhibitors, such as sorafenib, pazopanib, and imatinib, are the most common molecularly targeted drugs (7), with a phase III clinical trial of sorafenib vs. placebo revealing a total response rate of 33% in sorafenib group vs. 20% in the placebo group, as well as significant improvements in progression-free survival and remission duration (29). Other new drugs include γ-secretase inhibitors that target the Notch signaling pathway and provided a partial response rate of 29% and a disease stable rate of 29% (30).

During the last 10 years, there have been developments in various strategies for treating AF, with surgical treatment no longer being the only treatment choice due to its high long-term local recurrence. Nevertheless, only 1 of our 11 surgically treated patients experienced recurrence and that patient had undergone partial resection, which suggests that complete resection and negative margin are essential for reducing the long-term local recurrence rate. Furthermore, 2 patients received surveillance, and 3 patients received cytotoxic drug treatment, with these patients obtaining satisfactory efficacy and no surgery-related trauma. Thus, less invasive treatment strategies may be useful in future clinical practice.

## Conclusions

Intra-abdominal AF is a benign tumor with a high recurrence rate and an unclear pathogenesis. Surgery remains an effective technique for reducing the tumor burden and relieving symptoms. And negative margin are critical for reducing the risk of local recurrence rate. During the last decade, a systemic therapy model has been recommended for managing AF treatment, and physicians may wish to consider the tumor's biological behavior characteristics, as well as the patients' general condition and tolerance of surgery, when determining a personalized treatment strategy.

## Data Availability Statement

The database used and/or analyzed during the current study are not publicly available (to maintain privacy) but can be available from the corresponding author on reasonable request.

## Author Contributions

BL designed the study. JX and JM collected and analyzed the data. JM wrote the paper. JX and BL revised the paper. All authors have contributed to and approved the final manuscript.

### Conflict of Interest

The authors declare that the research was conducted in the absence of any commercial or financial relationships that could be construed as a potential conflict of interest.

## Abbreviations

AF: aggressive fibromatosis
DTs: desmoid tumors
DF: desmoid-type fibromatosis
PUMCH: Peking Union Medical College Hospital
FAP: familial adenomatous polyposis
APC: adenomatous polyposis coli
CT: computed tomography
MRI: magnetic resonance imaging
T1WI: T1-weighted images
T2WI: T2-weighted images
DSA: digital subtraction angiography
NSAIDs: non-steroidal anti-inflammatory drugs
APC: adenomatous polyposis coli.
